# Psychological distress and academic self-perception among international medical students: the role of peer social support

**DOI:** 10.1186/s12909-014-0256-3

**Published:** 2014-11-28

**Authors:** Yukari Yamada, Miloslav Klugar, Katerina Ivanova, Ivana Oborna

**Affiliations:** Department of Social Medicine and Public Health, Faculty of Medicine and Dentistry, Palacký University Olomouc, Hnevotinska 3, Olomouc, 775 15 Czech Republic; Department of Obstetrics and Gynecology, Faculty of Medicine and Dentistry, Palacký University Olomouc, Hnevotinska 3, Olomouc, 775 15 Czech Republic

**Keywords:** Medical student, Psychological distress, Academic perception, Social support, Effect modification, Additive interaction

## Abstract

**Background:**

Psychological distress among medical students is commonly observed during medical education and is generally related to poor academic self-perception. We evaluated the role of peer social support at medical schools in the association between psychological distress and academic self-perception.

**Methods:**

An online survey was conducted in a medical degree program for 138 international students educated in English in the Czech Republic. The Medical Student Well-Being Index was used to define the students’ psychological distress. Perceived peer social support was investigated with the Multidimensional Scale of Perceived Social Support. Poor academic self-perception was defined as the lowest 30% of a subscale score of the Dundee Ready Education Environment Measure. Analyses evaluated the presence of additive interactions between psychological distress and peer social support on poor academic self-perception, adjusted for possible confounders.

**Results:**

Both psychological distress and low peer social support were negatively associated with poor academic self-perception, adjusted for local language proficiency and social support from family. Students with psychological distress and low peer social support had an odds ratio of 11.0 (95% confidence interval (CI): 2.1–56.6) for poor academic self-perception as compared with those without distress who had high peer social support. The presence of an additive interaction was confirmed in that the joint association was four times as large as what would have been expected to be on summing the individual risks of psychological distress and low peer social support (synergy index = 4.5, 95% CI: 1.3–14.9).

**Conclusions:**

Psychological distress and low peer social support may synergistically increase the probability of poor academic self-perception among international medical students. Promoting peer social relationships at medical school may interrupt the vicious cycle of psychological distress and poor academic performance.

## Background

There is a general notion that many medical students experience psychological distress [1–3]. Psychological distress during medical education deserves serious attention because it is associated with poor academic performance [4,5], cynicism [6,7], an unwillingness to care for the chronically ill [8,9], and decreased empathy [9–11], all of which affect the quality of care provided by future physicians. However, completely removing stressors at medical school may be neither practical nor desirable [12]; therefore, medical schools are increasingly required to participate in promoting students’ resilience to distress [13,14].

Poor academic performance is one of the most worrisome issues intimately related to psychological distress. Several cross-sectional studies have repeatedly demonstrated associations between poor academic performance and psychological distress [5,15,16], though the direction of the association is difficult to determine [17]. Several longitudinal studies have in fact implied both directions. For example, students’ depression at enrolment predicted clinical competence measured 2 years later [18], whereas the second year’s stress levels were predicted by academic performance at earlier points even after controlling for stress levels at baseline [19]. Examining possible factors that could alter this negative association could have important implications for medical schools in the development of effective measures to improve student’s well-being and academic performance [20].

A theory suggests that students could be protected from the negative effects of stress through social support [21]. This is called the buffering hypothesis, wherein psychosocial stress will have deleterious effects on the health and well-being of those with little or no social support, whereas these effects will be diminished or eliminated for those with stronger support systems [22–27]. Cohen *et al.*, by defining combinations of particular types of stressors and social support, have proposed a model for a possible mechanism in which social support presumably has a buffering effect [22]. Among the types of social support described by Cohen *et al.* (i.e. tangible, appraisal, and emotional [self-esteem, belonging] support), appraisal and emotional support appear to be relevant to medical students experiencing poor academic performance and psychological distress. When one experiences psychological distress as a stressor, appraisal support can be effective because it could alter either one’s assessment of threat or one’s assessment of one’s ability to cope. By contrast, when one experiences poor academic performance as a stressor, which can result in negative feelings about one’s self, emotional support elevating one’s level of self-esteem is presumably needed. For both types of social support, an optimal source of such support could come from similar others who have experienced, or are experiencing, the same or similar situations [22].

On the basis of these analyses, we hypothesized that social support, particularly from peer friends at medical schools, could protect medical students from the vicious cycle of psychological distress and poor academic performance. This has not been rigorously studied in the literature, however. Our study therefore evaluated how peer social support could modify the association between psychological distress and poor academic performance in an international medical school.

## Methods

### Participants

Participants were medical students attending a medical degree program exclusively for foreigners and taught in English at the Palacky University in Olomouc, Czech Republic (hereafter, international medical students). There were 235 international medical students at the end of 2012; 58% were Malaysian, 26% British, and 11% Taiwanese. Other nationalities were represented by fewer than 5% of the students.

### Data collection

Detailed methods of the data collection in the international medical program are described elsewhere [28]. Briefly, we invited all international medical students (from the 1st year to the 6th year) to complete a web-based survey in December 2012. Participation was voluntary, and responses were anonymous. Students gave their consent to participate in the survey by starting the online questionnaire. Among the 235 students, 154 completed the survey, entailing an overall response rate of 66%. The distribution of respondents’ countries of origin was almost identical with that of all students in the program. The present study included 138 students who responded to all the questions regarding psychological distress, social support, and academic self-perception (response rate, 59%). No significant differences were observed in the students’ characteristics (i.e. sex, study year, marital status, language proficiencies, psychological distress, social support, and academic self-perception) between the 138 students and those excluded from the analyses. The study complied with the Declaration of Helsinki and was approved by the Institutional review Board; Etická komise Fakultuní nemocnice Olomouc a Lékařské fakulty UP v Olomouci.

### Psychological distress

Psychological distress is seldom defined as a distinct concept and is often embedded in the context of strain, stress, and distress. The Medical Student Well-Being Index (MSWBI) [29] has been developed to identify medical students in severe psychological distress. Accordingly, we used this instrument, with permission from the developers (the Mayo Foundation for Medical Education and Research). The MSWBI comprises seven items encompassing the domains of burnout (emotional exhaustion and depersonalization), depression, stress, fatigue, mental quality of life (QOL), and physical QOL. All questions are answered using a simple yes/no. One point is assigned for each ‘yes’, and summary scores of the seven-item index have a range of 0–7 (lowest to highest risk for severe distress). Satisfactory psychometric properties of the MSWBI have been shown [29]. At a threshold score of ≥4, the sensitivity and specificity for identifying students with low mental QOL or recent suicidal ideation/serious thoughts of dropping out were both ≥90%, and the prevalence of a false-negative score (score <4 in students with low mental QOL, suicidal ideation, or serious thoughts of dropping out) was estimated as being 5–7% [30]. In the present study, MSWBI scores were both used as a continuous scale and dichotomized into the ‘distressed’ and ‘not distressed’ categories, using ≥4 as the threshold.

### Social support

Peer social support is defined as social support from other students at a given medical school. We investigated this through a subscale of the Multidimensional Scale of Perceived Social Support (MSPSS) [31]. The MSPSS is a 12-item scale used to separately assess a subject’s perception of support from family, friends, and significant others. Each item is scored on a seven-point Likert scale (1 = very strongly disagree to 7 = very strongly agree). Scores on these three sources of support correspond to the MSPSS subscales of family support (MSPSS-FAM), friend support (MSPSS-FRI), and significant others support (MSPSS-SO), each of which accounts for 4–28 points. It has proven to be a psychometrically sound instrument, with good levels of internal consistency in several studies [32–34]. For the present study, we added a note that statements about friends apply to their current university life so that the MSPSS-FRI scores can be assumed to measure peer social support at medical school. MSPSS-FRI scores were used both as a continuous variable and as categorized by distinguishing the lowest 30% (low friend support), the middle (middle friend support), and the highest 30% (high friend support). To facilitate interpretation, the continuous scale was reversed in multivariate analyses so that a higher score indicated lower support. The MSPSS-FAM and MSPSS-SO were also analyzed separately as possible covariates that affect the relationship between peer social support and academic self-perception.

### Academic performance

Because the questionnaire was anonymous and objective data of academic performance were not available for this study, academic self-perception to indicate students’ perceptions of their academic performance was used as a proxy of academic performance. A subscale of the Dundee Ready Education Environment Measure (DREEM) was used for this purpose. The DREEM was developed to assess the educational climate in undergraduate medical and other health professional schools [35,36], primarily for diagnostic purposes [37]. Academic self-perception is one of the DREEM subscales. It has eight items including ‘I feel I am being prepared for my profession’, ‘I am confident about passing this year’, and ‘I am able to memorize all I need’ on a five-point Likert scale (0 = strongly disagree and 4 = strongly agree); accordingly, academic self-perception scores from 0 to 32, with a higher score indicating better perception. In the present study, students were classified as having ‘poor’ academic self-perception if they were within the lowest 30% of the distribution. Otherwise, the students were classified as having ‘not poor’ academic self-perception. Sensitivity analyses with other cut-off points, such as 20% with the lowest and the median distribution, were conducted. The above three measurements are summarized in Table 1.

**Table 1:** **Measurements used in this study**

**Variables**	**Measurements**	**Number of items**	**Range of score**	**Definition**
**Psychological distress**	Medical Student Well-Being Index (MSWBI)	7	0-7	Distress was defined as ≥4
**Social support**	Multidimensional Scale of Perceived Social Support (MSPSS)	12	12-84	-
Support from friends (= Peer social support)	MSPSS-FRI	4	4-28	Continuous
				Categorical: High (the highest 30%), middle, low (the lowest 30%)
Support from family	MSPSS-FAM	4	4-28	Continuous
Support from significant others	MSPSS-SO	4	4-28	Continuous
**Academic self-perception**	Subscale of Dundee Ready Education Environment Measure (DREEM)	8	0-32	Poor academic self-perception was defined as the lowest 30%

### Language proficiency

Language proficiencies in English and the local language were investigated with four possible answers of ‘native or native level’, ‘quite well’, ‘fair’, and ‘poor’. Both language proficiencies were dichotomized according to their distributions. English proficiency was categorized by distinguishing those who were ‘native or native level’ from the other categories. Czech proficiency was classified by distinguishing ‘poor’ from the other categories.

### Analysis

Possible covariates were sex, study year (preclinical = 1st to 3rd vs. clinical = 4th to 6th), marital status (married or engaged vs. others), language proficiency in English (native vs. non-native) and in the local language (some vs. poor), and social support from family and significant others. The only variables associated with poor academic performance were included as possible confounders in the subsequent analyses.

Analyses evaluated whether psychological distress and peer social support produced an additive interaction on academic self-perception. Logistic regression was performed with academic self-perception as the dependent variable using *STATISTICA* version 9 (StatSoft, Inc.). We first estimated the odds ratios of psychological distress and peer social support for poor academic self-perception separately. A combined variable was then made to see separate effects of the two exposures (i.e. low peer support and psychological distress), as well as the joint effects of the two exposures compared with the unexposed group as a joint reference category. To show the presence of an additive interaction, the Synergy Index (SI) was used. The SI is interpreted as the excess risk from exposure to both exposures when there is an interaction relative to the risk from exposure without an interaction: SI = (odds ratio for joint exposure to both risk factors −1)/([odds ratio for one risk factor – 1] + [odds ratio for other risk factor – 1]) [38]. In the absence of an interaction effect, SI equals 1. The 95% confidence interval (CI) for SI was computed using the recommended formulas [39].

## Results

Table 2 shows participants’ characteristics according to their own academic self-perception. Almost half (43%) of participating students were classified as distressed and there was a marginal association indicating that there were more distressed students among those who perceived their academic performance to be poor than among those who did not (p = 0.064). Students with poor academic self-perception had a higher MSWBI score (p <0.001) and a lower MSPSS-FRI score (p = 0.001). Among the possible covariates, local language proficiency and social support from family (MSPSS-FAM) were associated with academic self-perception; thus, they were included in the subsequent multivariate analyses.

**Table 2:** **Basic characteristics for academic self-perception (n = 138; Olomouc, the Czech Republic, 2012)**

	**Total**	**Academic self-perception**		***P*** **value** ^**1)**^
		**Poor**	**Not poor**	
	N = 138	N = 48	N = 90	
Gender, male, %	41	44	39	0.580
Study year, preclinical, %	32	31	32	0.907
Marital status, married or engaged, %	10	10	10	0.938
English, native or native level, %	33	35	31	0.607
Local language, poor, %	41	54	33	0.018
MSWBI^2)^, mean (SD)	3.2 (1.7)	3.9 (1.6)	2.9 (1.6)	<0.001
Psychological distress, %	43	54	38	0.064
MSPPS_FRI^3)^, mean (SD)	20.7 (5.5)	18.6 (5.5)	21.8 (5.2)	0.001
Low support from friends, %	27	44	18	0.004
Middle support from friends, %	35	29	38	
High support from friends, %	38	27	44	
MSPPS_FAM^4)^, mean (SD)	22.8 (5.7)	21.0 (6.3)	23.8 (5.2)	0.006
Low support from family, %	25	42	17	0.006
Middle support from family, %	41	31	46	
High support from family, %	34	27	38	
MSPPS_SO^5)^, mean (SD)	20.9 (5.9)	19.8 (6.6)	21.4 (5.4)	0.132
Low support from significant other, %	29	40	23	0.119
Middle support from significant other, %	30	23	33	
High support from significant other, %	41	38	43	

^1)^t-tests for means and the chi square tests for proportions.

^2)^Medical Student Well-Being Index (0–7): higher scores indicate higher distress.

^3)^Multidimensional Scale of Perceived Social Support from friends (4–28): higher scores indicate higher support from friends.

^4)^Multidimensional Scale of Perceived Social Support from family (4–28): higher scores indicate higher support from family.

^5)^Multidimensional Scale of Perceived Social Support from significant others (4–28): higher scores indicate lower support from significant other.

SD = standard deviation.

Table 3 shows the separate effects of exposures (i.e. low peer support and psychological distress) on poor academic self-perception, adjusted for local language proficiency and social support from family. Students with low peer support were more likely to perceive their academic performance to be poor as compared with those with high peer support (adjusted odds ratio (OR) = 3.6; 95% CI: 1.4–9.0). One score decrease in the MSPSS-FRI (i.e. less peer support) was associated with a higher OR of 1.1 (95% CI: 1.0–1.2), whereas one score increase in the MSWBI scale (i.e. more distress) was associated with a higher OR of 1.5 (95% CI: 1.1–1.9) for poor academic self-perception.

**Table 3:** **The odds ratio and 95% confidence intervals of social support from friends and psychological distress for poor academic self-perception among 138 international medical students (Olomouc, the Czech Republic, 2012)**

	**N (cases)**	**Crude OR (95% CI)**	**Adjusted OR (95% CI)** ^**1)**^
**Social support from friends**			
High	53 (13)	1	1
Middle	48 (14)	1.27 (0.52–3.06)	1.08 (0.44–2.68)
Low	37 (21)	**4.04 (1.64–9.96)**	**3.59 (1.43–9.00)**
Reversed MSPSS-FRI (continuous)^2)^		**1.11 (1.04–1.19)**	**1.10 (1.03–1.19)**
**Psychological distress**			
Not distress	78 (22)	1	1
Distressed	60 (26)	1.95 (0.96–3.96)	1.79 (0.83–3.85)
MSBWI (continuous)^3)^		**1.48 (1.17–1.87)**	**1.46 (1.13–1.89)**

^1)^Adjusted for local language proficiency, social support from family (continuous).

^2)^Reversed Multidimensional Scale of Perceived Social Support from friends (4–28): higher scores indicate lower support from friends.

^3)^Medical Student Well-Being Index (0–7): higher scores indicate higher distress.

OR: Odds ratio, CI: Confidence interval, significant odds are showed in bold.

**Figure 1:**
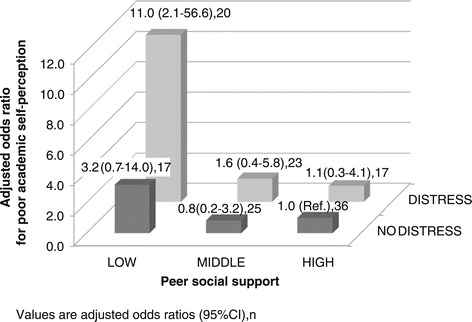
**Adjusted odds ratios for poor academic self-perception by peer social support and psychological distress.** The odds ratio for poor academic self-perception associated with the combination of peer social support and psychological distress, adjusted for local language proficiency and social support from family. Values are adjusted odds ratios (95% confidence intervals), n. Synergy index is 4.5 (96% confidence interval = 1.3-14.9).

Figure 1 shows the joint association between psychological distress and low peer support on poor academic self-perception, adjusted by local language proficiency and family support. Of the 20 students with psychological distress and low peer support, 65% perceived their academic performance as poor, which corresponds to an odds ratio of 11.0 (95% CI: 2.1–56.6), compared with the students without distress who had high peer support. SI for the joint association of psychological distress and low peer support was 4.5 (95% CI: 1.3–14.9), implying that the joint association was four times as large as what would have been expected to be for summing the individual risks of psychological distress and low peer social support.

We performed sensitivity analyses with different cut-off points of poor academic self-perception. We defined the lowest 20% and the median as alternatives and confirmed that all results indicated the same directions of association as the results presented in Tables 2 and 3 and Figure 1, with different degrees of significances (data not shown).

## Discussion

In this study, we presented results based on a cross-sectional single-site survey of international medical students studying medicine in English in a non-English speaking country. We found a high rate of psychological distress among the students and a strong association with poor academic self-perception, which is fairly consistent with previous studies in other settings [3–5]. The present study adds evidence that there was an interaction effect between low peer support and psychological distress on poor academic self-perception. Our findings may imply that promoting peer relationships at medical school could play an important role in protecting students from the vicious cycle of psychological distress and poor academic performance.

The protective and adaptive functions of positive peer relationships have been demonstrated across several areas of psychology [40–42], but with regard to medical students to the best of our knowledge no such study has been published. Rospenda *et al.*, however, reported findings seemingly contrary to our study; specifically, that in a university setting there were no social support buffering effects on the association between psychological distress and academic performance [43]. Two possible reasons could account for these different results. First, the study by Rospenda *et al.* did not specify “social support from within the university” to be peer support. Instead, such social support was likely to include support from teachers or the faculty rather than the peer students. According to the buffering theory to which we referred, peer friend support is more likely to have a buffering effect on the stress that medical students might experience. Second, differences in students’ characteristics might have had an impact. The students enrolled in the Rospenda *et al.* study were general medical students. It appears reasonable to assume that social support from friends at the university plays a more important role among international students whose social network is likely to be limited within the school [44], compared with local students who are likely to have other local social networks.

We focused on social support from friends in this study, because we presumed this to theoretically be an optimal source of appraisal and emotional support. However, other sources of social support could also have a buffering effect if they offer the same types of support. In fact, family support is a possible source, as it had a positive association with better academic self-perception. Family support remained significant in the multivariate analyses (e.g. the odds ratio of inversed MSPSS-FAM for poor academic self-perception in Figure 1 was 1.1 (95% CI: 1.0–1.2), which implies that family support was associated with academic self-perception, independent of friend support. In contrast, social support from significant others was not likely to have a buffering effect; it was not associated with academic self-perception. This finding is in line with Rospenda *et al.*’s findings that, among women, higher levels of social support outside of medical school were associated with worse academic performance [43]. This finding may be also related to our previous findings that medical students having frequent contact with significant others experienced significant psychological distress [28]. The role of social support from significant others for medical students needs to be investigated in further studies.

Additionally, we found a positive association between local language proficiency and academic self-perception. This finding may merely indicate a possible strong relationship between one’s ability to gain a new language and academic performance as a medical student. It could also indicate that acquiring some of a local language may actually benefit a student’s academic self-perception, because it is the local language that is required in their clinical practice as well as in daily life. Therefore, greater emphasis on teaching the local language might have a positive influence on medical students in this particular setting.

Of practical concern is what medical schools can do to enhance their students’ peer relationships. To create an atmosphere that promotes positive peer relationships, schools should not neglect the social aspects of a student’s program. A study conducted among early adolescents illustrates this [45]. The study was based on a combination of the social interdependence theory and relevant experimental studies and argued that more positive peer relationships (e.g. mutual help and assistance, sharing resources and information, and acting in a trustworthy and trusting manner) were associated with a cooperative rather than competitive or individualistic goal structure. A cooperative goal structure indicates that the goals of separate individuals are so linked together that there is a positive correlation between their attainment of goals. Accordingly, applied to the medical school arena, medical schools could tie the passing of an exam to other benefits, for example.

There are several important limitations to our findings. First, the study is cross-sectional. Although we have indicated a possible protective effect of social support from friends on the association between psychological distress and academic self-perception, it is not possible to draw firm conclusions. Longitudinal associations should be investigated to speculate on potential mechanisms by which social support from friends might intervene in the associations between distress and academic self-perception. Second, generalizability of our findings should be limited to international medical students in a specialized English medical school in non-English speaking countries. However, it might be possible to apply our findings to general medical students by using a more strict definition of peer social support. Third, the measurement we used to quantify academic self-perception has to be validated. We used this because objective measures of academic performance were not available for our study. Although the subscale of academic self-perception has been shown to have good internal consistency [46], validation of this subscale against objective measures of academic performance would strengthen our findings. Last, some important variables that could have had an influence on the association of interest in this study may have been omitted, such as the financial status and personal traits of each student. Further study is needed to investigate the possible influence of these factors.

## Conclusions

Psychological distress and low peer social support synergistically may increase a probability for poor academic self-perception among international medical students. Promoting peer social relationships at medical school may interrupt the vicious cycle of psychological distress and poor academic performance.

## Abbreviations

MSWBI: The medical student well-being index
MSPSS: The multidimensional scale of perceived social support
MSPSS-FAM: A subscale of MSPSS “Social support from family”
MSPSS-FRI: A subscale of MSPSS “Social support from friends”
MSPSS-SO: A subscale of MSPSS “Social support from significant others”
DREEM: The dundee ready education environment measure
